# The Antimicrobial Effect of *Melissa officinalis* L. Essential Oil on *Vibrio parahaemolyticus*: Insights Based on the Cell Membrane and External Structure

**DOI:** 10.3389/fmicb.2022.812792

**Published:** 2022-03-10

**Authors:** Huijie Yu, Juxin Pei, Weiqiang Qiu, Jun Mei, Jing Xie

**Affiliations:** ^1^College of Food Science and Technology, Shanghai Ocean University, Shanghai, China; ^2^Shanghai Professional Technology Service Platform on Cold Chain Equipment Performance and Energy Saving Evaluation, Shanghai Ocean University, Shanghai, China; ^3^National Experimental Teaching Demonstration Center for Food Science and Engineering, Shanghai Ocean University, Shanghai, China; ^4^Shanghai Engineering Research Center of Aquatic Product Processing and Preservation, Shanghai Ocean University, Shanghai, China

**Keywords:** *Melissa officinalis* L., *Vibrio parahaemolyticus*, antimicrobial mechanism, biofilm, virulence genes

## Abstract

The study was to evaluate the antimicrobial impacts on *Melissa officinalis* L. essential oil (MOEO) against *Vibrio parahaemolyticus*. The minimum inhibitory concentration (MIC) of MOEO on *Vibrio parahaemolyticus* was 1 μL⋅mL^–1^. The kill-time curve exhibited that MOEO had good antimicrobial activity. The analysis of cellular ingredients leakage and cell viability illustrated that MOEO has destruction to the morphology of the cell membrane. The damage to the membrane integrity by MOEO has been confirmed by transmission and scanning electron microscopy, obvious morphological and ultrastructural changes were observed in the treated bacterial cells. The MOEO at 0.5 μL⋅mL^–1^ can inhibit the biofilm formation, biofilm motility, and extracellular polysaccharide production. Meanwhile, the qPCR results exhibited MOEO inhibited the expression of virulence genes. The findings showed that MOEO exerted its antimicrobial effect mainly by destroying the membrane, which indicated its potential as a natural food preservative.

## Introduction

*Vibrio parahaemolyticus* can lead to vibriosis in different species of aquatic animals, along with septicemia and gastroenteritis in humans (Ning et al., 2021). It is the most common *Vibrio* genus and has recently become a primary food safety issue in many Asian countries (Zhu et al., 2022). *V. parahaemolyticus* is recognized as a new species because of vibriosis related to the consumption of contaminated raw or undercooked seafood (Ashrafudoulla et al., 2021). Sepsis has also been reported when wounds were exposed to the pathogen (Zhong et al., 2021).

Adding natural preservatives are the common methods to control the growth and reproduction of *V. parahaemolyticus* (Semeniuc et al., 2017). Essential oils (EO), a complex mixture of aromatic and volatile consisting secondary metabolites of aromatic plants, is usually obtained from plant material such as flowers, fruits, and leaves (Spadaccino et al., 2021). *Melissa officinalis* L. (MO) is a plant that has been used to give fragrance to foods and beverages. As a medicinal plant, it has been given various therapeutic roles (anticonvulsant, energizer, sedative, etc.) (Serra et al., 2020). *Melissa officinalis* L. essential oil (MOEO) primarily contains terpene aldehydes (citronellal, citral, and geranial) and terpenic alcohols (dimethylocta-2, dimethyl-3-octanol, etc.) (Rãdulescu et al., 2021), which demonstrated good potential for antimicrobial activity.

Biofilms on microorganisms are referred to as a dense network structure made up of exopolysaccharides (EPS) and multifarious microorganisms embedded in them (Popławski et al., 2008). Biofilms may form on medical devices, the surfaces of food, and the equipment to transport raw materials. The biofilm environment acts just like a physical barricade and drives both metabolic and physiological changes, adapting microorganisms to a slow growth and situation of starvation, and increasing their resistance to various preservatives (Sahal et al., 2020). The purpose of the study was to detect the possible mechanism of antimicrobial action that *V. parahaemolyticus* treated by MOEO. The effect of *V. parahaemolyticus* treated by MOEO was decided by electric conductivity, glucose content, field emission Fourier transform infrared (FTIR) spectroscopic analysis, transmission electron microscope (TEM), field emission scanning electron microscope (SEM), biofilm formation, and the virulence genes expression.

## Materials and Methods

### Gas Analysis by Gas Chromatography-Mass Spectrometry

The analysis of MOEO is carried out by Gas Chromatography-Mass Spectrometry (GC-MS) (Trace DSQ II, United States). A DB-5H capillary fused silica column was used. The temperatures of injector and detector were adjusted to 250 and 230°C, correspondingly. The ion source temp was set to 230°C. The oven temperature was maintained at 45°C for 1 min, followed by adjustment to 300°C at a speed of 20°C/min. The injection sample volume was 1 μL, the ionization energy was 70 EV, and the scan range was 35–500 m/z. Identification of MOEO was a comparison of retention times and mass spectral libraries.

### Chemicals and Bacterial Culture

MOEO was purchased from Tokyo Chemical Industry Co., Ltd. (Tokyo, Japan). *V. parahaemolyticus* ATCC 33847 was used in this study. There was 100 μL stock culture inoculated into 10 mL TSB medium (Hopebio, Qingdao) containing 3% NaCl (w/v). The strain was propagated in TSB medium (Hopebio, Qingdao) containing 3% NaCl (w/v) at 37°C with shaking at 200 r/min to receive the initial culture which concentration was 10^8^ CFU/mL. MOEO was dissolved with 5% Tween-80 (v/v) proportionally, and then sonicated for 30 min until completely dissolved.

### Minimum Inhibitory Concentration and Minimum Bactericidal Concentration Measurements

The MIC and MBC of *V. parahaemolyticus* treated with MOEO were ensured by the broth microdilution method according to the slightly modified method from Wang L. et al. (2020). There were 96-well microtiter plates added with the diluted bacterial culture at a concentration of 1 × 10^8^ cfu/mL. Serial dilutions of MOEO were made up and the mixture was added to each well with final concentration range from 0.125 to 256 μL/mL. All plates were followed 24 h incubation at 37°C, then assessed for growth by turbidimetric method. Defined MIC as the lowest MOEO concentration that no visible growth was detected (OD600 ≤ 0.05). To evaluate the bacterial growth further, the cultures with no visible bacteria growth were subcultured on nutrient agar to determine that the minimum concentration of MOEO was defined as MBC, with no colony growth (Liu W. et al., 2020).

### Kill-Time Analysis

To study the effect of *V. parahaemolyticus* growth kinetics by MOEO treated, we used the kill-time assay according to a former research report with some modifications (Guo et al., 2019). *V. parahaemolyticus* was treated with different concentrations of MOEO (2×, 1×, 0.5× and 0.25× MIC) and 5% Tween-80. The culture cultured at 37°C and monitored OD_600_. The experiments were carried out in triplicate.

### Measurements of Electric Conductivity and Glucose Content

The electric conductivity was measured based on Chen et al. (2021) with some modifications. *V. parahaemolyticus* was treated with MOEO 10 h at 37°C, then the mixture was centrifugated. The supernatant was diluted 40-fold and measured the conductivity every 2 h with an electrical conductivity facility (CN121-A, Nuclear Instrument Co., Ltd., China). The glucose content was determined every 2 h with a glucose assay kit (No. 361510, Jiancheng, Nanjing, China).

### Integrity of Cell Membrane

MOEO with the range of concentration ranges (2×, 1×, 0.5× and 0.25× MIC) was added to the initial culture (1 × 10^8^ cfu/mL), then the mixed culture was shaking incubated 4 h at 37°C. The supernatant was collected by centrifugating at 8,000 × g, 10 min at 4°C. Using a UV spectrophotometer (UV-2100, UNICO Instrument Co., Ltd., China), we monitored nucleic acid and protein levels, respectively (Wang N. et al., 2020).

### Scanning Electron Microscope Analysis

MOEO with the range of concentration ranges (2×, 1×, 0.5×, and 0.25× MIC) were added to the initial culture (1 × 10^8^ cfu/mL), then the mixed culture was shaking incubated 4 h at 37°C. The culture was subjected to centrifugation to collect the bacteria cells. The *V. parahaemolyticus* samples were fixed in 2.5% glutaraldehyde solution over 4 h and cleaned with 0.01 mol/L PBS solution. The *V. parahaemolyticus* samples were then eluted with ethanol. After air-drying on coverslips, the sediment was sputtered with gold and imaged with SEM (S-3400, Hitachi, Tokyo, Japan).

### Transmission Electron Microscope Analysis

MOEO with the range of concentration ranges (1× MIC) were added to the initial culture (1 × 10^8^ cfu/mL), then the mixed culture (1 × 10^8^ cfu/mL), then the mixed culture was shaking incubated 4 h at 37°C. The culture was subjected to centrifugation to collect the bacteria cells. The *V. parahaemolyticus* samples were fixed in 2.5% glutaraldehyde solution over 4 h. The following treatments refer to Tan et al. (2018), using a JEM-2100 TEM (JEOL Ltd., Japan) observed.

### Fourier Transform Infrared Spectroscopic Analysis

MOEO with the range of concentration ranges (2×, 1×, 0.5×, and 0.25× MIC) were added to the initial culture (1 × 10^8^ cfu/mL), then the mixed culture was shaking incubated 4 h at 37°C. The culture was subjected to centrifugation to collect the bacteria cells. The *V. parahaemolyticus* samples were fixed in 2.5% glutaraldehyde solution over 4 h and cleaned with 0.01 mol/L PBS three times. The sediment was freeze-dried for 48 h and then subjected to FTIR spectroscopy (Nicolet, Thermo Fisher Scientific, United States) (Li et al., 2016).

### Crystal Violet Quantitative Assay

The influence of MOEO on biofilm was assessed indirectly by crystal violet staining (Zhang et al., 2017a). MOEO with the range of concentration ranges (2×, 1×, 0.5×, and 0.25× MIC) was added to the initial culture (1 × 10^8^ cfu/mL), and then cultured in a 48-well plate for 48 h, the presence of biofilm growth was assayed. After 24 h cultured in TSB, the cell culture was seeded to another plate in TSB and allowed to adhere. The suspending cells were then aspirated and 200 μL of fresh TSB with MOEO was added, incubated for a further 24 h, and then measured the biofilm production. The supernatant was removing floating cells, washing, and drying twice, the biofilm was stained with crystal violet. The solutions were taken to a new well and the absorbance was sensed at 570 nm. The experimental procedure was carried out in triplicate.

### Motility Assay

The impact of MOEO on the motility assay were assessed swimming and swarming. For swarming motility assay, 5 μL of 1 × 10^8^ CFU/mL *V. parahaemolyticus* bacterial culture was added to the LB dishes with 1.5% (w/v) agar with MOEO at concentrations of 0.5× and 0.25× MIC, incubating at 37°C for 24 h. To the swimming motility assay, 5 μL of 1 × 10^8^ CFU/mL bacterial culture was added to the center of the LB dishes with 0.3% (w/v) agar with MOEO at concentrations of 0.5× and 0.25× MIC, incubating at 37°C for 12 h.

### Exopolysaccharides Assay

MOEO with the range of concentration ranges (0.5× and 0.25× MIC) was added to the initial culture (1 × 10^8^ cfu/mL) and cultured in a 48-well plate 6 h at 37°C in an anaerobic environment to observe the amount of biofilm. The effects of MOEO were explored through the phenol-sulfuric acid (PSA) method (Cao et al., 2021).

### Confocal Laser Scanning Microscopy Assay

MOEO with the range of concentration ranges (0.5× and 0.25× MIC) was added to the initial culture and cultured in a 48-well plate 24 h at 37°C under anaerobic condition. We visualized the effect of MOEO on biofilm using confocal laser scanning microscopy (CLSM). After 24 h of biofilm formation, we removed the culture, and cleaned plastic panels three times with 0.1 M PBS. The 48-well plates were fixed with glutaraldehyde, and then the plastic panel was rinsed three times with 0.1 M PBS to eliminate glutaraldehyde. Subsequently, plastic panels were stained with SYBR Green I (Sangon Biotech, Co., Ltd., Shanghai, China) under dark conditions for 30 min. The plastic panels used 0.1 M PBS cleaned to remove extra stain. Last, the biofilm was studied with a CLSM (LSM710, Carl Zeiss AG, Germany) at 488 nm excitation light and 525 ± 25 nm emitting light, following a 20 × microscope objective (Jun and Jing, 2020).

### Quantitative Realtime PCR

MOEO (0.5 × and 0.25 × MIC) was added to the initial culture, oscillation cultured 6 h, then the culture was centrifuged to collect the bacteria cells. The total RNA was extracted. Of the total RNA, 1,000 ng, 1 μL of the primers oligo (dT), and 1 μL of 10 mmol/L dNTP mix was used to obtain the cDNA. MA-6000 Real Time PCR (Suzhou Yarui Biotechnology Co., Ltd., China) was used for quantitative realtime PCR (qPCR) assay. The relative expression levels were counted using the comparative threshold cycle (2^–ΔΔCt^) method, and gapdh was defined as the reference gene. The gene sequences are in Table 1.

**TABLE 1:** The sequence of primers.

Target gene	Sequence of primers (5′–3′)
	
Trh	F, ACGCGGTTGATGTTCGTAGT
	R, ACCTCATATCATCGCGCAGG
vopS	F, CTGGCAAGAACCTCAAAGCG
	R, GCCCTTCAATATGTCGCTGC
vscF	F, GAGCAACAAGCGAAAGACGC
	R, AGTGGTGGTTGCGTTGATGT
VP1388	F, CAACATCAAACATGCCGCGT
	R, AGCTACACGCATGTCCCTTC
VP1407	F, TCTTACGAGCGTAGTTGGCG
	R, CGGCTGATGAAGTACAACCG
toxR	F, TGAACCAGAAGCGCCAGTAG
	R, TTGTCCGCCAGTGGCAATTA
toxS	F, CCCGTTACGTCGTGTGAATG
	R, TTGTCGATTCAGCCGTCGAG
opaR	F, AGCTCGATCATCGCATTGGT
	R, TCAACCATGTTGTCCGTCAGT
aphA	F, GGCTTGTGCTGTTCAACCAT
	R, GTTACGACGAAGCGTTAGGC
luxM	F, ACCTGAGGTCAGTTCATGCTT
	R, TTCCGTTCCTGCGTGTCC
luxS	F, GCAGGGTTTGACTCCACACT
	R, TGATGGCTGCTGCAATGAGT
luxN	F, CAAACTCGGCGGGCATTGAT
	R, GGACGACGCAAAAGATCCTC
luxU	F, TTTCGGAGCCGACAGTTTGT
	R, CGTCGCGTGTTTCATTCAAG
luxO	F, GCGTCATGGCTCTCAAGACT
	R, TAGCGGCAGAGTCAATGGTG

### Statistical Analysis

In all experiments, three simultaneous parallel dimensions were carried out. Statistical analysis was conducted with the software SPSS 22.0. Results are expressed as mean ± SD. A *P*-value of 0.05 or less was a difference that was statistically significant.

## Results and Discussion

### Gas Chromatography-Mass Spectrometry Analyses

The compositions of MOEO were determined by GC-MS, and 11 main components were identified and quantified (Table 2). The main active constituents of MOEO were geraniol, citronella, and citronellol. Geraniol constituted 38.31% of the total amount. Citronella constituted 27.87% of the total amount, and was the second volatile component in MOEO, followed by citronellol (11.38%). The results were similar with the research reported by Miraj et al. (2017). MOEO was found in the presence of numerous phytochemicals such as phenolic components (tannins, flavonoids, and phenolic acids) and terpenes (triterpenes, sesquiterpenes, and monoterpenes) (Ilić et al., 2021). Miraj et al. (2017) found the predominant character of MOEO was present as oxygen-containing monoterpenes such as geranial, citronellal, and citral isomers which were the antimicrobial ingredient. Actually, the main ingredients of MOEO, citronellal and geranial, have been shown to be in charge of its antimicrobial activity which were proved to be responsible for its antimicrobial activity (Božović et al., 2018). Neda et al. (2004) found that the citral and citronellol content were 12 and 13%, respectively. Also, the MOEO showed a better antimicrobial effectiveness. The difference of MOEO levels was likely caused by different growing environments or different extraction methods (Caleja et al., 2017; Silva et al., 2021).

**TABLE 2:** The formulation of MOEO.

No.	Compounds	RT (min)	PA (%)
1	D-limonene	4.74	4.13
2	3,7-dimethylocta-1,6-dien-3-ol	5.335	1.1
3	Citronellal	5.815	27.87
4	Citronellol	6.4	11.38
5	Geraniol	6.6	38.31
6	β-Citronellol	7.335	2.75
7	Eugenol	7.415	1.05
8	Geranyl acetate	7.555	7.42
9	β-elemene	7.73	1.96
10	D-iso macrogeradiene	8.425	1.24
11	α-copaene	8.64	2.79

### Minimum Inhibitory Concentration and MBC of *Melissa officinalis* L. Essential Oil Against *Vibrio parahaemolyticus*

**FIGURE 1:**
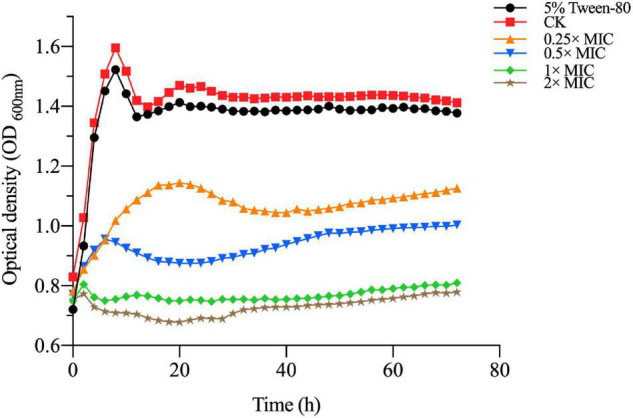
The *V. parahaemolyticus* growth curves. CK is the *V. parahaemolyticus* without *Melissa officinalis* L. essential oil treated.

The MIC and MBC of MOEO against *V. parahaemolyticus* were 1 and 2 μL/mL, correspondingly. To further prove the antimicrobial activity of MOEO against *V. parahaemolyticus*, the influence of *V.* parahaemolyticus growth in the existence of levels (0.25×, 0.5×, 1×, 2× MIC and 5% Tween-80) were plotted. In Figure 1, there was no obvious difference between the growth value of 5% Tween-80 and CK. This result was consistent with Orafidiya et al. (2001) and illustrated 5% Tween-80 was not antimicrobial to *V. parahaemolyticus* (Rizvi et al., 2013). The increase of *V. parahaemolyticus* was inhibited with different concentrations of MOEO and the effect was a dose-dependent and time-dependent manner. This result was consistent with Chen et al. (2021). There was no obvious difference between the growth value of 1 × MIC and 2 × MIC, and the growth of *V. parahaemolyticus* was completely controlled. These findings indicated that MOEO had an antimicrobial effect on *V. parahaemolyticus* and increased with the dose. The antimicrobial activities of MOEO may probably be due to the citronellol, geranial, and D-limonene. Geranial and citronellal have been implicated in the antimicrobial and antifungal activities of the EO (Božović et al., 2018).

### Cell Membrane Permeability

**FIGURE 2:**
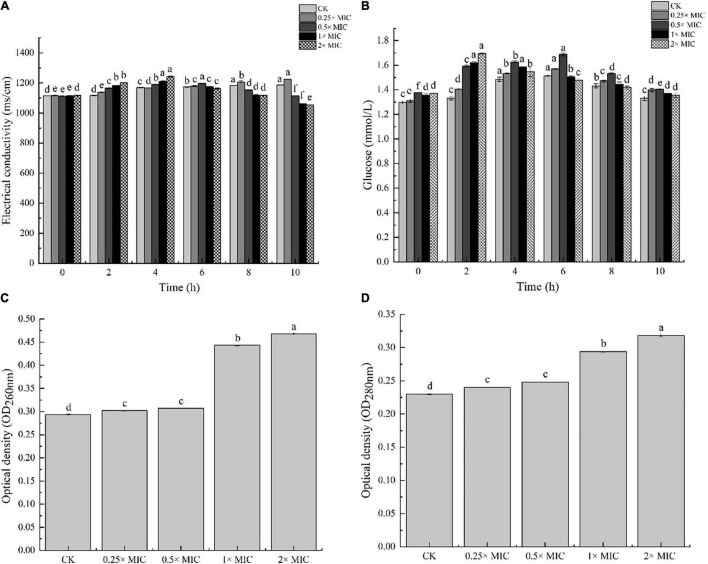
The effects of electric conductivity **(A)**, and intracellular glucose spillage **(B)**, nucleic acids **(C)** and proteins **(D)** from *V. parahaemolyticus* treated with *Melissa officinalis* L. essential oil. CK is the *V. parahaemolyticus* without *Melissa officinalis* L. essential oil treated.

The experiment of MOEO about electric conductivity was carried out to determine, in a greater degree, the disruption of cell architecture. In Figure 2A, the leakage degree was positively correlated with the treated time at 0.25 × MIC MOEO. The electrical conductivity values of *V. parahaemolyticus* with MOEO treatment at levels of 1 × MIC and 2 × MIC were significantly increased after 2 h and decreased after 2 h. The cell membrane was destroyed and small molecules of Na^+^ and K^+^ leaked from the cells. Fluctuations in the conductivity of the control may be caused by autolysis and bacterial cell death (Zhang et al., 2017b). The immediate increase of the culture electrical conductivity upon the addition of the MOEO can be attributed to the high reactivity and volatility of the natural essential oil (Cui et al., 2016; Zhang et al., 2018). The mentioned phenomena were consistent with the results of TEM analysis, which gave further evidence of the disruption caused by MOEO to the cell membrane and wall of *V. parahaemolyticus*.

### Cell Membrane Integrity

Nucleic acids, proteins, and glucose were the essential substances presenting throughout the cell membrane and cytoplasm of bacteria, and they flowed out as the bacterial membrane was damaged (Xu et al., 2018). Therefore, the leakage of these substances can reflect the integrity of the membrane. In Figure 2B, the amount of extracellular glucose also went up with concentration, and the impact was time dependent. Conductivity values of *V. parahaemolyticus* by 1 × MIC MOEO treated increased obviously from 0 to 2 h and decreased after 4 h. The trend was the same at 2 × MIC concentrations, which can be owned to the stress response of mycelium (Zhang et al., 2018). As shown in Figures 2C,D, nucleic acids and proteins leaked from *V. parahaemolyticus* cells after the treatment of MOEO, the effect was dose related. There was no obvious difference between OD_260_ and OD_280_ value of 0.25 × MIC and 0.5 × MIC with the control. The nucleic acids and proteins treated with 0.25 × MIC MOEO increased by 1.47, 1.25 times compared to control, respectively. These results were consistent with Dai et al. (2021) that they found *Litsea cubeba* essential oils disrupted the integrity of *Escherichia coli* and caused the leakage of nucleic acids and proteins. This finding suggested that MOEO destroyed the cell membrane leading to the increase in extracellular proteins and nucleic acids.

### Electron Microscopic Observations

**FIGURE 3:**
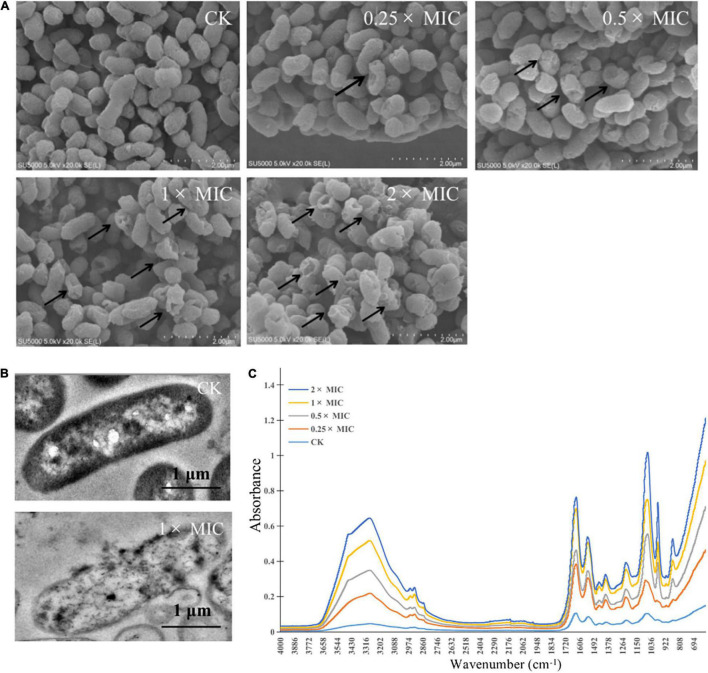
Effects of *Melissa officinalis* L. essential oil on *V. parahaemolyticus* shown in SEM [**(A)** The magnification is 50,000×], TEM **(B)** images, and the FTIR spectroscopy **(C)**. CK is the *V. parahaemolyticus* without *Melissa officinalis* L. essential oil treated.

The morphological alterations of the cells and spores treated with different concentrations of MOEO (2×, 1×, 0.5×, and 0.25× MIC, respectively) were observed by SEM. In Figure 3A, the control showed rod-shaped cells with intact morphology and smooth surface, the cell had intact and dense protoplasm. In contrast, the cell structure changed significantly after treatment with different concentrations of MOEO. Irregular and distinct wrinkled with pucker and small holes appeared on the surface of *V. parahaemolyticus* treated with 0.25 × MIC MOEO. More cells became distorted and shriveled in 0.5 × MOEO treatment. The 1 × and 2 × MIC MOEO treated samples caused more severe membrane damage and the surface had many dents and wrinkles. Together, these results were consistent with the conclusions above, confirming that the bacterial cell membrane was damaged by treatment with MOEO. A similar change of *Shewanella putrefaciens* in SEM images after phenyllactic acid treated was reported (Fang et al., 2021). Ferreira et al. (2011) found that the *Pseudomonas fluorescens* appeared rougher with wrinkles and membrane deformed after citral treatment. All these results indicated that MOEO treatment disrupted the cell walls and cell membranes of the bacteria at different levels. Cells were disrupted and divided as a result of the loss of cell integrity.

Morphological changes of *V. parahaemolyticus* after treatment with MOEO (1 × and 0 × MIC, respectively) were observed by TEM. Cellular secretions in treated cells were found to leak in the surrounding medium by TEM studies (Guo et al., 2018). The controlling group kept the typical bacterial morphology with clear cytoplasm, cell wall, cell membrane, and nuclei (Figure 3B). Cells that were treated with 1 × MIC MOEO (Figure 3B) showed a marked variability. Bacterial cell membranes and walls were disrupted, lysed, and broken. In addition, the cytoplasm of the bacteria extravasated, appearing as a clear cavity, slightly deformed, cell wall broken, and cytoplasm leaked from the treated *V. parahaemolyticus*. Deng et al. (2021) demonstrated that anthocyanins inhibited the growth of *E. coli* by disrupting the bacterial cell wall and plasma membrane making cell dissolution and cytoplasmic released. Dai et al. (2021) reported that the strain cells treated with *Litsea cubeba* essential oil lead to disordered function of cell membranes and obvious intracellular injury appeared. Zhou et al. (2021) showed that treatment of *E. coli* with *Alpinia galanga rhizomes* essential oil can stimulate the efflux of intracellular material. Liu W. et al. (2020) interpreted that cell membranes and walls of *P. fluorescens* and *S. putrefaciens* treated with Daphnetin were disrupted and resolved, and the cytoplasm of the bacteria leaked, creating a clear cavity. Such phenomena suggested that adding polyphenols may disrupt cell division, leading to cells changing from classic long rod shape to short rod shape (Yi et al., 2010.). Apparently, the cells of strains that were treated with MOEO sustained major injury due to induced cell membrane dysfunction, according to the results (Chen et al., 2017).

### Fourier Transform Infrared Spectroscopic

Secondary structure was measured using the FTIR technique, with the purpose of exploring the antimicrobial effect of MOEO against *V. parahaemolyticus* (Figure 3C). The slight changes at 982 cm^–1^ were attributed to the cyclic oscillation of oligosaccharides and polysaccharides (Salman et al., 2019). The 1,185–1,485 cm^–1^ region bands were dominated by the protein, lipids, and phosphate compounds contribution, so the peak changed at 1,386 cm^–1^ and showed the growth of *V. parahaemolyticus* was inhibited by MOEO. Changes in the characteristic peaks of absorbed at 3,260 and 1,218 cm^–1^ showed that MOEO may destroy cell membrane phospholipid structure (Kos et al., 2003). The 1,080cm^–1^ is due to symmetric phosphate stretching modes (Fujioka et al., 2004), and the decrease is probably because of the leakage of carbohydrates, nucleic acids, and polysaccharides (Salman et al., 2019). The characteristic absorption bands reduced at 1,061 cm^–1^ also indicating the leakage of nucleic acids. Meanwhile, the characteristic absorption bands reduced at 1,629 cm^–1^ suggested that protein leaked. These discoveries were the same as the result of cell membrane integrity analysis.

### Effect of *Melissa officinalis* L. Essential Oil on *Vibrio parahaemolyticus* Biofilm Formation

**FIGURE 4:**
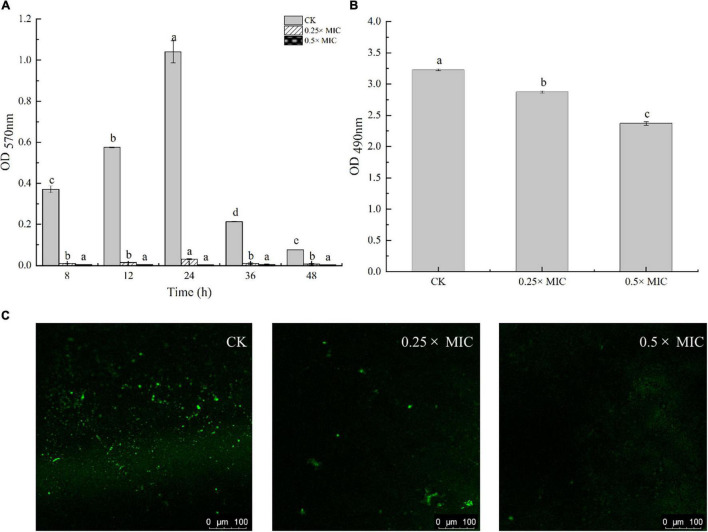
Effect of *Melissa officinalis* L. essential oil on biofilm formation **(A)**, EPS production **(B)**, and CLSM **(C)**. CK is the *V. parahaemolyticus* without *Melissa officinalis* L. essential oil treated.

Anti-biofilm action of MOEO was evaluated by a crystalline violet test. It was shown that MOEO prevented biofilm generation and the effect was in a dose dependent manner (Figure 4A). Biofilm quantities were reduced after subinhibitory concentrations (SICs) MOEO treated. There were 90.31 and 98.27% inhibition ratio in 0.25 × MIC and 0.5 × MIC MOEO treatments for 8 h, respectively, and no biofilm formed after 24 h. MOEO disturbed the biofilm formation from the initiation stage. As a result, MOEO demonstrated excellent suppression of biofilm formation.

**FIGURE 5:**
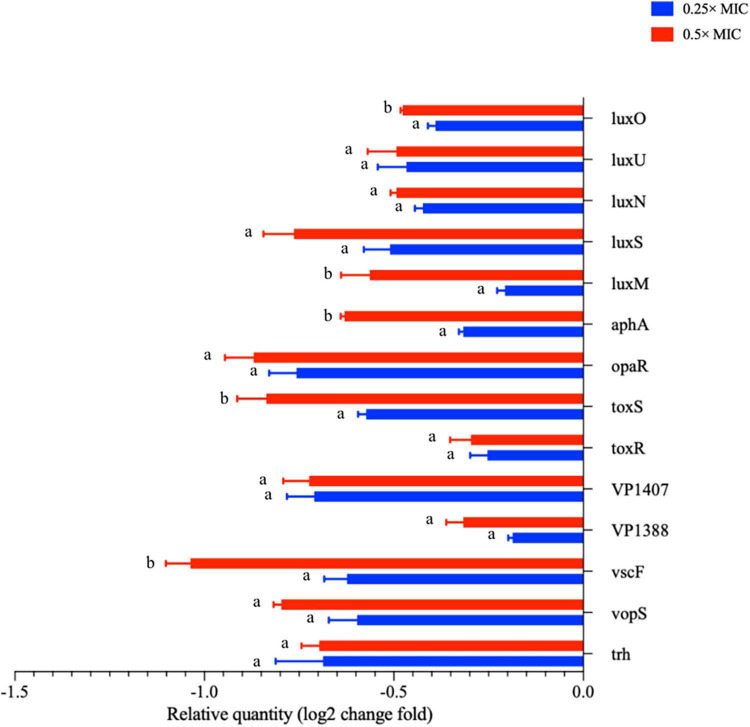
The transcription levels of virulence related genes of *V. parahaemolyticus* treated with *Melissa officinalis* L. essential oil. CK is the *V. parahaemolyticus* without *Melissa officinalis* L. essential oil treated.

Cao et al. (2021) and Ruan et al. (2021) found that citral essential oil and resveratrol can suppress the biofilm formation in *V. parahaemolyticus* and *E. coli*. The biofilm formation in the control group decreased at 36 h may arise from cell apoptosis cutting down the amounts of bacteria that can form biofilms. The antimicrobial effect of MOEO may be related to its refrained formation of biofilm. In addition to environmental factors, the formation of biofilm was also influenced by the control of bacterial quorum sensing system (QS) (He et al., 2019). A classic two-component signal transduction system, the Lux system in Vibrio species, had been shown to contribute to biofilms’ growth (Deborah and Groisman, 2013). Liu et al. (2021) demonstrated that LuxQ, LuxU, and LuxO were critical for biofilm formation control in *V. parahaemolyticus* and the deletion mutants among them displayed analogous biomembrane-forming. It can be concluded that MOEO inhibited the expression of *LuxQ*, *LuxU*, and *LuxO*, thus reducing the formation of biofilms (Figure 5).

### Inhibition of Motility by *Melissa officinalis* L. Essential Oil

**FIGURE 6:**
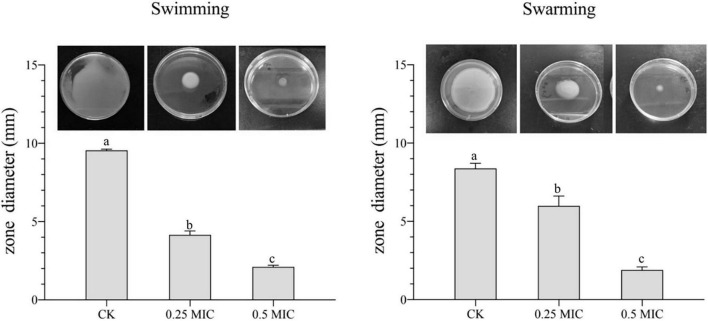
Effects of *Melissa officinalis* L. essential oil on the motility of *V. parahaemolyticus.* CK is the *V. parahaemolyticus* without *Melissa officinalis* L. essential oil treated.

In Figure 6, MOEO decreased swimming and swarming motilities of *V. parahaemolyticus* with a dose-dependent effect. With increasing MOEO concentration, the colony’s diameters of *V. parahaemolyticus* reduced. Swimming motility of *V. parahaemolyticus* was markedly suppressed at 0.25 × MIC compared to the control. When the concentration was 0.5 × MIC, smaller colonies of bacteria were observed, illustrating higher inhibition of swimming motility. In addition, the active effect of MOEO to control swarming motility was also shown. The swarming motility of bacteria was suppressed by 0.25 × and 0.5 × MIC MOEO. As the diameter of bacterial colonies decreased, bacterial colonies progressively grew thinner. Jin et al. (2021) revealed that the garlic essential oil prevented the motility of Bacillus cereus ATCC 14579, and this experiment has indicated that MOEO could also restrict swarming and swimming motility of *V. parahaemolyticus*. The ability to swarm enabled *V. parahaemolyticus* cells to move through the environment, making it possible for them to get a suitable surface to stick to Jin et al. (2021).

### Exopolysaccharides Production

Adhering to a solid surface, bacteria allowed bacteria to keep growing and secreting a matrix of biofilm outside the polymer, which included nucleic acids, proteins, and EPS. Extracellular polymeric matrix constituted 80% of the biofilm and formed a mature biofilm architecture. EPS is one of the main constituents of the extrapolymeric matrix. Therefore, it is important to suppress or decrease the production of EPS to control the biofilm formation (Liu F. et al., 2020).

Detection of EPS level in *V. parahaemolyticus* biofilms by quantification assay. In Figure 4B, adding 0.25 × and 0.5 × MIC MOEO markedly suppressed EPS contents in *V. parahaemolyticus* biofilms and EPS production decreased 4.87 and 17.48% in 0.25 × MIC and 0.5 × MIC MOEO treated samples, respectively. Thus, treatment with 0.25 × and 0.5 × MIC MOEO can suppress EPS production of *V. parahaemolyticus* cells in biofilms. The decrease of EPS was consistent with the result of the decreased biofilm production. Extracellular polymers of biofilms played an important part during the initial stages of adherence and biofilm formation by bacteria (Colagiorgi et al., 2016). Bacterial biofilms gave bacteria cells protection against harsh environments (Stoodley et al., 2002), and the inhibition of MOEO on *V. parahaemolyticus* growth can also be explained.

### Confocal Microscopy Observations (Confocal Laser Scanning Microscopy)

In Figure 4C, *V. parahaemolyticus* cells are not exposed (a, control) and exposed to 0.25 × and 0.5 × MIC MOEO. The amount of biofilm production was observed by treating the biofilm with SYBR Green I (Morozov et al., 2021). The results showed that the formation of biofilm matrix can be controlled by MOEO. According to CLSM images, biofilms were tightly attached to each other, and more cells fluoresced green in the visual field. In MOEO treated samples, biofilm structure was thin and shape appeared turbid. Compared with other groups, hardly any green in the visual field with 0.5 × MIC MOEO treated, showing that bacteria leaked the extrapolymeric matrix in the biofilm. These above phenomena were consistent with crystal violet quantitative assay.

### *Melissa officinalis* L. Essential Oil Suppressed the Virulence Gene Presentation

Under the influence of MOEO, virulence factor-associated genes in *V. parahaemolyticus* were displayed in Figure 5. The regulator gene T3SS (*vopS*, *vscF*, and *trh*), T6SS (*VP1388* and *VP1407*), ToxRS system (*toxR* and *toxS*), and the relation genes of QS (*luxU*, *luxS*, *luxN*, *luxO*, *luxM*, *opaR*, and *aphA*) were obviously lowered due to the treatment of MOEO. A greater suppression has been observed at 0.5 × MIC compared to 0.25 × MIC.

QS molecules have been shown to be implicated in the forming of bacterial biofilms (Rodolfo et al., 2016). *V. parahaemolyticus* mainly exploited the classical LuxI/LuxR system to create acyl homologous lactones (AHL) as signaling molecules to QS. AHL facilitated the formation of biofilm in *V. parahaemolyticus* (Ding et al., 2018). In Figure 5, it can be seen that MOEO controlled the expression of *luxM*, *luxS*, *luxN*, *luxU*, *luxO*, and *opaR* that were connected with QS. Liu et al. (2021) built the double deletion strains ΔluxUΔluxQ and ΔluxUΔluxO and assessed the number of biofilms, the result suggested that *luxQ*, *luxO*, and *luxU* were linked in the same signaling route regulating *V. parahaemolyticus* biofilm formation. Regarding additional *Vibrio* species, a recent study indicated that *Vibrio cholerae* used the Lux pathway by which to regulate biofilm formation (Jung et al., 2015). Ray and Visick (2012) proposed that *luxQ* exerted its function through *luxU* to regulate biofilm formation in *Vibrio fischeri. LuxU* controlled the transcription of syp loci (symbiotic polysaccharides) through the SypG-dependent pathway and *luxO.* Ray and Visick (2012) concluded that the Lux route influenced the formation of biofilms with *luxU* and *luxU* had a greater effect on biofilm formation than *luxO*, probably because the functions of *luxU* cognate appeared diverse in different *Vibrio* species. In general, Lux systems can affect the formation of biofilms in *Vibrio* species.

D-limonene, linalool, and citronellol were the main components of MOEO, which have been shown to inhibit the QS system of bacteria (Deborah and Groisman, 2013; Ding et al., 2018; Zhang et al., 2018; He et al., 2019; Dai et al., 2021; Liu et al., 2021; Zhou et al., 2021). D-limonene can inhibit the activity of signaling molecules by degrading them or reducing their production (He et al., 2019). Linalool was able to reduce the activity of signal molecule synthase or signal molecule receptor protein and block the QS pathway (Stewart and McCarter, 2003). In addition, *opaR* can encode the key regulator at high cell density (Deborah and Groisman, 2013), *tdh* was the particular element of virulent *V. parahaemolyticus* strain (Liu et al., 2021). They were all inhibited by the gene expression.

Previous study on this bacterium revealed that T3SS system and T6SS system assisted the existence and multiplication of the bacterium in the human gastrointestinal tract. Furthermore, some effector proteins were produced by these secretion systems that were involved in immunosuppression, cytotoxicity, and dysregulation to the actin network (Burdette et al., 2008; Hubbard et al., 2016). Xiaohui et al. (2010) discovered that *VP1686* secreted from T3SS was accountable for the cytotoxicity of the host cell. Toshio et al. (2008) identified *vopD2* and *vopB2*, two effector proteins under T3SS, in charge of pore formation and cytotoxicity from infected cells. After treatment with MOEO, the relation genes of T3SS system and T6SS system expression were downmodulated, which may cause suppression of biofilm generation and virulence.

ToxRS is a chromosomally encoded gene with the primary function of regulating the virulence factors expression. The two-component regulator ToxRS proved to be critical to bacterial persistence and virulence of *V. parahaemolyticus* at the time of host infection (Yiquan et al., 2018). This study revealed that MOEO significantly repressed the *toxR* and *toxS* genes transcription from a dose-dependent manner.

## Conclusion

This research investigated the antimicrobial activity of MOEO against *V. parahaemolyticus* and its mechanism. The MIC of MOEO action on *V. parahaemolyticus* was 1 μL⋅mL^–1^. MOEO disrupted cell wall and membrane integrity, resulting in nucleic acid and protein efflux. SEM, TEM, and CLSM outcomes illustrated that MOEO altered the morphology of bacterial cells, allowing the efflux of materials in bacteria. FTIR spectra revealed that MOEO broke the phospholipid structure on the membrane, leading to nucleic acid and protein efflux, inhibiting the *V. parahaemolyticus* growth. At SICs, MOEO reduced the quantity of biofilm, slackened the biofilm structure, inhibited motility, as well as significantly reduced its potential virulence. MOEO impeded the process of QS in *V. parahaemolyticus*, downmodulated the regulatory genes T3SS, T6SS, and ToxRS system and the relational genes of QS to inhibit biofilm production. Those results demonstrated that MOEO can reduce the virulence risk even if the MOEO concentrations in food or packaging have not reached MIC for *V. parahaemolyticus*. Thus, MOEO is a promising natural preservative for the food industry, becoming a viable solution to decrease microbial growth.

## Data Availability Statement

The original contributions presented in the study are included in the article/supplementary material, further inquiries can be directed to the corresponding author/s.

## Author Contributions

HY: conceptualization, methodology, software, investigation, and writing. JP: methodology and investigation. WQ: conceptualization and software. JM: validation, formal analysis, writing—review, and editing. JX: examination and funding acquisition. All authors contributed to the article and approved the submitted version.

## Conflict of Interest

The authors declare that the research was conducted in the absence of any commercial or financial relationships that could be construed as a potential conflict of interest.

## Publisher’s Note

All claims expressed in this article are solely those of the authors and do not necessarily represent those of their affiliated organizations, or those of the publisher, the editors and the reviewers. Any product that may be evaluated in this article, or claim that may be made by its manufacturer, is not guaranteed or endorsed by the publisher.

## References

[B1] Ashrafudoulla M. Na K. W. Hossain M. I. Mizan M. F. R. Nahar S. Toushik S. H. (2021). Molecular and pathogenic characterization of Vibrio parahaemolyticus isolated from seafood. *Mar. Pollut. Bull.* 172:112927. 10.1016/j.marpolbul.2021.112927 34526263

[B2] Božović M. Garzoli S. Baldisserotto A. Romagnoli C. Pepi F. Cesa S. (2018). Melissa officinalis L. subsp. altissima (Sibth. & Sm.) Arcang. essential oil: chemical composition and preliminary antimicrobial investigation of samples obtained at different harvesting periods and by fractionated extractions. *Ind. Crops Prod.* 117 317–321. 10.1016/j.indcrop.2018.03.018

[B3] Burdette D. L. Yarbrough M. L. Orvedahl A. Gilpin C. J. Orth K. (2008). Vibrio parahaemolyticus Orchestrates a Multifaceted Host Cell Infection by Induction of Autophagy, Cell Rounding, and Then Cell Lysis. *Proc. Natl. Acad. Sci. U.S.A.* 105 12497–12502. 10.1073/j.pnas.2008.0802773105 18713860 PMC2527940

[B4] Caleja C. Barros L. Prieto M. A. Barreiro M. F. Oliveira M. B. P. P. Ferreira I. C. F. R. (2017). Extraction of rosmarinic acid from Melissa officinalis L. by heat-, microwave- and ultrasound-assisted extraction techniques: a comparative study through response surface analysis. *Sep. Purif. Technol.* 186 297–308. 10.1016/j.seppur.2017.06.029

[B5] Cao J. Liu H. Wang Y. He X. Jiang H. Yao J. (2021). Antimicrobial and antivirulence efficacies of citral against foodborne pathogen Vibrio parahaemolyticus RIMD2210633. *Food Control* 120:107507. 10.1016/j.foodcont.2020.107507

[B6] Chen F. Miao X. Lin Z. Xiu Y. Shi L. Zhang Q. (2021). Disruption of metabolic function and redox homeostasis as antibacterial mechanism of Lindera glauca fruit essential oil against *Shigella* flexneri. *Food Control* 130:108282. 10.1016/j.foodcont.2021.108282

[B7] Chen M. Zhao Z. Meng H. Yu S. (2017). The antibiotic activity and mechanisms of sugar beet (Beta vulgaris) molasses polyphenols against selected food-borne pathogens. *LWT Food Sci. Technol.* 82 354–360. 10.1016/j.lwt.2017.04.063

[B8] Colagiorgi A. Ciccio P. D. Zanardi E. Ghidini S. Ianieri A. (2016). A Look inside the Listeria monocytogenes Biofilms Extracellular Matrix. *Microorganisms* 4:22. 10.3390/j.microorganisms.2016.4030022 PMC5039582 27681916

[B9] Cui H. Li W. Li C. Vittayapadung S. Lin L. (2016). Liposome containing cinnamon oil with antibacterial activity against methicillin-resistant Staphylococcus aureus biofilm. *Biofouling* 32 215–225. 10.1080/j.bio.2015.08927014.1134516 26838161

[B10] Dai J. Li C. Cui H. Lin L. (2021). Unraveling the anti-bacterial mechanism of Litsea cubeba essential oil against E. coli O157:H7 and its application in vegetable juices. *Int. J. Food Microbiol.* 338:108989. 10.1016/j.ijfoodmicro.2020.108989 33257098

[B11] Deborah C. H. Groisman E. A. (2013). The Biology of the PmrA/PmrB Two-Component System: the Major Regulator of Lipopolysaccharide Modifications. *Ann. Rev. Microbiol.* 67 83–112. 10.1146/annurev-micro-092412-155751 23799815 PMC8381567

[B12] Deng H. Zhu J. Tong Y. Kong Y. Tan C. Wang M. (2021). Antibacterial characteristics and mechanisms of action of Aronia melanocarpa anthocyanins against *Escherichia coli*. *LWT* 150:112018. 10.1016/j.lwt.2021.112018

[B13] Ding T. Li T. Li J. (2018). Identification of natural product compounds as quorum sensing inhibitors in *Pseudomonas fluorescens* P07 through virtual screening. *Bioorg. Med. Chem.* 26, 4088–4099. 10.1016/j.bmc.2018.06.039 30100021

[B14] Fang M. Wang R. Agyekumwaa A. K. Yu Y. Xiao X. (2021). Antibacterial effect of phenyllactic acid against Vibrio parahaemolyticus and its application on raw salmon fillets. *LWT* 112586 [preprint] 10.1016/j.lwt.2021.112586

[B15] Ferreira C. Pereira A. M. Pereira M. C. Melo L. F. Simões M. (2011). Physiological changes induced by the quaternary ammonium compound benzyldimethyldodecylammonium chloride on *Pseudomonas* fluorescens. *J. Antimicrob. Chemotherapy* 66 1036–1043. 10.1093/j.jac/dkr028 21393196

[B16] Fujioka N. Morimoto Y. Arai T. Kikuchi M. (2004). Discrimination between normal and malignant human gastric tissues by Fourier transform infrared spectroscopy. *Cancer Detect. Prev.* 28 32–36. 10.1016/j.cdp.2003.11.004 15041075

[B17] Guo J. Gao Z. Li G. Fu F. Liang Z. Zhu H. (2019). Antimicrobial and antibiofilm efficacy and mechanism of essential oil from Citrus Changshan-huyou Y. B. chang against Listeria monocytogenes. *Food Control* 105 256–264. 10.1016/j.foodcont.2019.06.014

[B18] Guo Z.-Y. Zhang Z.-Y. Xiao J.-Q. Qin J.-H. Zhao W. (2018). Antibacterial Effects of Leaf Extract of Nandina domestica and the Underlined Mechanism. *Evid. Based Complementary Altern. Med.* 2018:8298151. 10.1155/2018/8298151 29576801 PMC5822912

[B19] He L. Le K. Y. Khan B. A. Nguyen T. H. Hunt R. L. Bae J. S. (2019). Resistance to leukocytes ties benefits of quorum sensing dysfunctionality to biofilm infection. *Nature Microbiol.* 4 1114–1119. 10.1038/j.naturemicr.s41564-019-0413-x 30936487 PMC6588452

[B20] Hubbard T. P. Chao M. C. Abel S. Blondel C. J. Abel Zur Wiesch P. Zhou X. (2016). Genetic analysis of Vibrio parahaemolyticus intestinal colonization. *Proc. Natl. Acad. Sci. U.S.A.* 113 6283–6288. 10.1073/pnas.1601718113 27185914 PMC4896720

[B21] Ilić Z. S. Milenković L. Tmušić N. Stanojević L. Stanojević J. Cvetković D. (2021). Essential oils content, composition and antioxidant activity of lemon balm, mint and sweet basil from Serbia. *LWT* 153:112210. 10.1016/j.lwt.2021.112210 PMC8568991 34764769

[B22] Jin Z. Li L. Zheng Y. An P. (2021). Diallyl disulfide, the antibacterial component of garlic essential oil, inhibits the toxicity of Bacillus cereus ATCC 14579 at sub-inhibitory concentrations. *Food Control* 126:108090. 10.1016/j.foodcont.2021.108090

[B23] Jun Y. Jing X. (2020). Comparative Proteome Analysis of Shewanella putrefaciens WS13 Mature Biofilm Under Cold Stress. *Front. Microbiol.* 11:1225. 10.3389/fmicb.2020.01225 32582122 PMC7296144

[B24] Jung S. A. Chapman C. A. Wai-Leung N. (2015). Quadruple quorum-sensing inputs control *Vibrio cholerae* virulence and maintain system robustness. *PLoS Pathog.* 11:e1004837. 10.1371/j.ppat.2015.1004837 25874462 PMC4398556

[B25] Kos G. Lohninger H. Krska R. (2003). Development of a method for the determination of Fusarium fungi on corn using mid-infrared spectroscopy with attenuated total reflection and chemometrics. *Anal. Chem.* 75 1211–1217. 10.1021/j.ac.2003.0260903 12641243

[B26] Li H. Gao Y. Li C. Ma G. Shang Y. Sun Y. (2016). A comparative study of the antibacterial mechanisms of silver ion and silver nanoparticles by Fourier transform infrared spectroscopy. *Vib. Spectrosc.* 85 112–121. 10.1016/j.vibspec.2016.04.007

[B27] Liu F. Jin P. Gong H. Sun Z. Du L. Wang D. (2020). Antibacterial and antibiofilm activities of thyme oil against foodborne multiple antibiotics-resistant Enterococcus faecalis. *Poult. Sci.* 99 5127–5136. 10.1016/j.psj.2020.06.067 32988551 PMC7598324

[B28] Liu M. Zhu X. Zhang C. Zhao Z. (2021). LuxQ-LuxU-LuxO pathway regulates biofilm formation by Vibrio parahaemolyticus. *Microbiol. Res.* 250:126791. 10.1016/j.micres.2021.126791 34090181

[B29] Liu W. Mei J. Xie J. (2020). Elucidating Antibacterial Activity and Mechanism of Daphnetin against *Pseudomonas* fluorescens and Shewanella putrefaciens. *J. Food Quality* 2020 1–10. 10.1155/j.foodq.2020.6622355

[B30] Miraj S. Rafieian-Kopaei Kiani S. (2017). Melissa officinalis L: a Review Study With an Antioxidant Prospective. *J. Evid. Based Complementary Altern. Med.* 22 385–394. 10.1177/j.2017.2156587216663433 27620926 PMC5871149

[B31] Morozov V. N. Kolyvanova M. A. Dement’eva O. V. Rudoy V. M. Kuzmin V. A. (2021). Comparison of quenching efficacy of SYBR Green I and PicoGreen fluorescence by ultrasmall gold nanoparticles in isotropic and liquid-crystalline DNA systems. *J. Mol. Liquids* 321:114751. 10.1016/j.molliq.2020.114751

[B32] Neda M.-D. Biljana B. Marina S. Natasa S. (2004). Antimicrobial and antioxidant activities of Melissa officinalis L. (Lamiaceae) essential oil. *J. Agricult. food chem.* 52 2485–2489. 10.1021/jf030698a 15113145

[B33] Ning H. Cong Y. Lin H. Wang J. (2021). Development of cationic peptide chimeric lysins based on phage lysin Lysqdvp001 and their antibacterial effects against Vibrio parahaemolyticus: a preliminary study. *Int. J. Food Microbiol.* 358:109396. 10.1016/j.ijfoodmicro.2021.109396 34560361

[B34] Orafidiya L. O. Oyedele A. O. Shittu A. O. Elujoba A. A. (2001). The formulation of an effective topical antibacterial product containing Ocimum gratissimum leaf essential oil. *Int. J. Pharm.* 224 177–183. 10.1016/S0378-5173(01)00764-5 11472827

[B35] Popławski N. J. Abbas S. Maciej S. Glazier J. A. (2008). Simulation of single-species bacterial-biofilm growth using the Glazier-Graner-Hogeweg model and the CompuCell3D modeling environment. *Mathematical biosciences and engineering*. *MBE* 5 355–388. 10.3934/j.mbe.2008.5.355 18613738 PMC2547990

[B36] Rãdulescu M. Jianu C. Lukinich-Gruia A. T. Mioc M. Mioc A. Şoica C. (2021). Chemical Composition, In Vitro and In Silico Antioxidant Potential of Melissa officinalis subsp. *Officinalis Essential Oil*. *Antioxidants* 10:1081. 10.3390/antiox10071081 34356313 PMC8301138

[B37] Ray V. A. Visick K. L. (2012). LuxU connects quorum sensing to biofilm formation in V ibrio fischeri. *Mol. Microbiol.* 86 954–970. 10.1111/mmi.12035 23035866 PMC3566283

[B38] Rizvi M. Ahmed J. Khan F. Shukla I. Malik A. (2013). Assessment of combination therapy by time kill curve analysis and chequerboard assay for treatment of multi-drug resistant *Pseudomonas aeruginosa* isolates. *J. Global Antimicrob. Resist.* 1 103–108. 10.1016/j.jgar.2013.04.001 27873576

[B39] Rodolfo G.-C. Toshinari M. Wood T. K. (2016). Can resistance against quorum-sensing interference be selected? *ISME J.* 10 4–10. 10.1038/j.ismej.2015.84 26023871 PMC4681860

[B40] Ruan X. Deng X. Tan M. Wang Y. Hu J. Sun Y. (2021). Effect of resveratrol on the biofilm formation and physiological properties of avian pathogenic *Escherichia coli*. *J Proteom.* 249:104357. 10.1016/j.jprot.2021.104357 34450330

[B41] Sahal G. Woerdenbag H. J. Hinrichs W. L. J. Visser A. Tepper P. G. Quax W. J. (2020). Antifungal and biofilm inhibitory effect of Cymbopogon citratus (lemongrass) essential oil on biofilm forming by Candida tropicalis isolates; an in vitro study. *J. Ethnopharmacol.* 246:112188. 10.1016/j.jep.2019.112188 31470085

[B42] Salman A. Shufan E. Sharaha U. Lapidot I. Mordechai S. Huleihel M. (2019). Distinction between mixed genus bacteria using infrared spectroscopy and multivariate analysis. *Vib. Spectros.* 100 6–13. 10.1016/j.vibspec.2018.10.009

[B43] Semeniuc C. A. Pop C. R. Rotar A. M. (2017). Antibacterial activity and interactions of plant essential oil combinations against Gram-positive and Gram-negative bacteria. *J. Food Drug Anal.* 25 403–408. 10.1016/j.jfda.2016.06.002 28911683 PMC9332530

[B44] Serra E. Saubade F. Ligorio C. Whitehead K. Sloan A. Williams D. W. (2020). Methylcellulose Hydrogel with Melissa officinalis Essential Oil as a Potential Treatment for Oral Candidiasis. *Microorganisms* 8:215. 10.3390/microorganisms8020215 32041100 PMC7074814

[B45] Silva T. C. Bertolucci S. K. V. Carvalho A. A. Tostes W. N. Alvarenga I. C. A. Pacheco F. V. (2021). Macroelement omission in hydroponic systems changes plant growth and chemical composition of Melissa officinalis L. essential oil. *J. Appl. Res. Med. Arom. Plants* 24:100297. 10.1016/j.jarmap.2021.100297

[B46] Spadaccino G. Frabboni L. Petruzzi F. Disciglio G. Mentana A. Nardiello D. (2021). Essential oil characterization of Prunus spinosa L., Salvia officinalis L., Eucalyptus globulus L., Melissa officinalis L. and Mentha x piperita L. by a volatolomic approach. *J. Pharm. Biomed. Anal.* 202:114167. 10.1016/j.jpba.2021.114167 34058537

[B47] Stewart B. J. McCarter L. L. (2003). Lateral flagellar gene system of Vibrio parahaemolyticus. *J. Bacteriol.* 185 4508–4518. 10.1128/j.jb.2003.185.15.4508-4518 12867460 PMC165745

[B48] Stoodley P. Sauer K. Davies D. G. Costerton J. W. (2002). BIOFILMS AS COMPLEX DIFFERENTIATED COMMUNITIES. *Ann. Rev. Microbiol.* 56 187–209. 10.1146/annurev.micro.56.012302.160705 12142477

[B49] Tan Z. Bo T. Guo F. Cui J. Jia S. (2018). Effects of ε-Poly-l-lysine on the cell wall of Saccharomyces cerevisiae and its involved antimicrobial mechanism. *Int. J. Biol. Macromol.* 118 2230–2236. 10.1016/j.ijbiomac.2018.07.094 30026097

[B50] Toshio K. Hirotaka H. Kazuyoshi G. Yukihiro A. Shigeaki M. Kwon-Sam P. (2008). Identification of two translocon proteins of Vibrio parahaemolyticus type III secretion system 2. *Infect. Immun.* 76 4282–4289. 10.1128/j.IAI.2008.01738-07 18541652 PMC2519421

[B51] Wang L. Zhang K. Zhang K. Zhang J. Fu J. Li J. (2020). Antibacterial Activity of Cinnamomum camphora Essential Oil on *Escherichia coli* During Planktonic Growth and Biofilm Formation. *Front. Microbiol.* 11:561002. 10.3389/fmicb.2020.561002 33304322 PMC7693543

[B52] Wang N. Liu X. Li J. Zhang Q. Li X. An Q. (2020). Antibacterial mechanism of the synergistic combination between streptomycin and alcohol extracts from the Chimonanthus salicifolius S. *Y. Hu. leaves*. *J. Ethnopharmacol.* 250:112467. 10.1016/j.jep.2019.112467 31837412

[B53] Xiaohui Z. Konkel M. E. Call D. R. (2010). Vp1659 is a Vibrio parahaemolyticus type III secretion system 1 protein that contributes to translocation of effector proteins needed to induce cytolysis, autophagy, and disruption of actin structure in HeLa cells. *J. Bacteriol.* 192 3491–3502. 10.1128/j.jb.01493-09 20418402 PMC2897656

[B54] Xu D. Sun L. Li C. Wang Y. Ye R. (2018). Inhibitory effect of glucose oxidase from Bacillus sp. *LWT Food Sci. Technol*. 92 339–346. 10.1016/j.lwt.2018.02.025

[B55] Yi S.-M. Zhu J.-L. Fu L.-L. Li J.-R. (2010). Tea polyphenols inhibit *Pseudomonas aeruginosa* through damage to the cell membrane. *Int. J. Food Microbiol.* 144 111–117. 10.1016/j.ijfoodmicro.2010.09.005 20884071

[B56] Yiquan Z. Lingfei H. George O. A. Ying Z. Wenhui Y. Zhe Y. (2018). Autoregulation of ToxR and Its Regulatory Actions on Major Virulence Gene Loci in Vibrio parahaemolyticus. *Front. Cell. Infect. Microbiol.* 8:291. 10.3389/j.fcimb.2018.00291 30234024 PMC6135047

[B57] Zhang N. Lan W. Wang Q. Sun X. Xie J. (2018). Antibacterial mechanism of Ginkgo biloba leaf extract when applied to Shewanella putrefaciens and Saprophytic staphylococcus. *Aquacul. Fish.* 3 163–169. 10.1016/j.aaf.2018.05.005

[B58] Zhang Y. Wang Y. Zhu X. Cao P. Wei S. Lu Y. (2017a). Antibacterial and antibiofilm activities of eugenol from essential oil of Syzygium aromaticum (L.) Merr. & L. M. Perry (clove) leaf against periodontal pathogen Porphyromonas gingivalis. *Microbial. Pathogenesis* 113 396–402. 10.1016/j.micpath.2017.10.054 29101062

[B59] Zhang Y. Wu Y.-T. Zheng W. Han X.-X. Jiang Y.-H. Hu P.-L. (2017b). The antibacterial activity and antibacterial mechanism of a polysaccharide from Cordyceps cicadae. *J. Fun. Foods* 38 273–279. 10.1016/j.jff.2017.09.047

[B60] Zhong X. Lu R. Liu F. Ye J. Zhao J. Wang F. (2021). Identification of LuxR Family Regulators That Integrate Into Quorum Sensing Circuit in Vibrio parahaemolyticus. *Front. Microbiol.* 12:691842. 10.3389/j.fmicb.2021.691842 34267739 PMC8276238

[B61] Zhou C. Li C. Siva S. Cui H. Lin L. (2021). Chemical composition, antibacterial activity and study of the interaction mechanisms of the main compounds present in the Alpinia galanga rhizomes essential oil. *Ind. Crops Products* 165:113441. 10.1016/j.indcrop.2021.113441

[B62] Zhu W. Gao J. Liu H. Liu J. Jin T. Qin N. (2022). Antibiofilm effect of sodium butyrate against Vibrio parahaemolyticus. *Food Control* 131:108422. 10.1016/j.foodcont.2021.108422

